# SSH2.0: A Better Tool for Predicting the Hydrophobic Interaction Risk of Monoclonal Antibody

**DOI:** 10.3389/fgene.2022.842127

**Published:** 2022-03-15

**Authors:** Yuwei Zhou, Shiyang Xie, Yue Yang, Lixu Jiang, Siqi Liu, Wei Li, Hamza Bukari Abagna, Lin Ning, Jian Huang

**Affiliations:** ^1^ Center for Informational Biology, University of Electronic Science and Technology of China, Chengdu, China; ^2^ School of Life Science and Technology, University of Electronic Science and Technology of China, Chengdu, China; ^3^ School of Healthcare Technology, Chengdu Neusoft University, Chengdu, China

**Keywords:** therapeutic antibody, developability, hydrophobic interactions, support vector machine, prediction model

## Abstract

Therapeutic antibodies play a crucial role in the treatment of various diseases. However, the success rate of antibody drug development is low partially because of unfavourable biophysical properties of antibody drug candidates such as the high aggregation tendency, which is mainly driven by hydrophobic interactions of antibody molecules. Therefore, early screening of the risk of hydrophobic interaction of antibody drug candidates is crucial. Experimental screening is laborious, time-consuming, and costly, warranting the development of efficient and high-throughput computational tools for prediction of hydrophobic interactions of therapeutic antibodies. In the present study, 131 antibodies with hydrophobic interaction experiment data were used to train a new support vector machine-based ensemble model, termed SSH2.0, to predict the hydrophobic interactions of antibodies. Feature selection was performed against CKSAAGP by using the graph-based algorithm MRMD2.0. Based on the antibody sequence, SSH2.0 achieved the sensitivity and accuracy of 100.00 and 83.97%, respectively. This approach eliminates the need of three-dimensional structure of antibodies and enables rapid screening of therapeutic antibody candidates in the early developmental stage, thereby saving time and cost. In addition, a web server was constructed that is freely available at http://i.uestc.edu.cn/SSH2/.

## Introduction

Antibodies play an indispensable role in the vertebrate immune defence system (Kapingidza et al., 2020). They also serve as essential agents in biomedical research and clinical diagnostic assays such as enzyme-linked immunosorbent assay, immunohistochemical assay, and immunoprecipitation assay. Furthermore, antibodies have been extensively used in clinical treatment of many types of cancers, autoimmune diseases, and infectious diseases including the coronavirus disease 2019, which is caused by the severe acute respiratory syndrome coronavirus 2 (Ning et al., 2021). Rapid development of the monoclonal antibody (mAb) technology has revolutionised pharmaceutical science and industry. Many proteins that cannot interact with small chemical molecules or are undruggable due to self-tolerance are considered efficient targets for antibody drugs. More than 550 therapeutic mAbs have been tested in phase I/II clinical trials worldwide, of which 79 mAbs have entered the final stage of development (Kaplon et al., 2020). Antibody drugs account for a large market share in the pharmaceutical industry. In 2018, the therapeutic antibodies had a global value of United States $115.2 billion, which is expected to reach $300 billion by the end of 2025 (Lu et al., 2020). Moreover, the large-scale application of antibody phage display, single B-cell antibody, and next-generation sequencing technologies has resulted in the development of tens of thousands of preclinical therapeutic antibody drug candidates. However, the probability of a human or humanised antibody drug candidate, which is under clinical trials, being approved is low (approximately 15%) (Carter and Lazar, 2018). Many mAbs fail due to unfavourable physicochemical properties such as high viscosity, increased aggregation tendency, and susceptibility to chemical degradation (Jain et al., 2017b).

Protein aggregation has been considered as one of the major challenges in biological drug development. It poses challenges during different developmental processes from fermentation and purification to storage (Obrezanova et al., 2015). It not only reduces the effectiveness of a drug but also induces adverse immune responses in patients (Martinez Morales et al., 2019). Thus, identifying therapeutic antibody candidates with high aggregation tendency at the early developmental stage is essential. The factors that affect protein aggregation are either intrinsic (e.g., interaction between hydrophobic patches, van der Waals forces and electrostatic interactions) or extrinsic (e.g., pH, salt concentration, buffer type, and storage conditions). Among these factors, the presence of hydrophobic moieties on the protein surface is the strongest determinant (Hebditch et al., 2019). A few tools to predict the hydrophobicity of proteins including mAbs have been reported (Lienqueo et al., 2006; Mahn et al., 2009; Hanke et al., 2016; Jain et al., 2017a). However, most of these tools rely on protein structures and do not provide free web services. In our previous study, we developed a tool called SSH, which can predict the hydrophobic interaction risk of mAbs solely by using the mAb sequences (Dzisoo et al., 2020). The SSH tool was trained with the tripeptide composition (TPC), and the prediction accuracy of 91.226% was achieved through the voting strategy. However, the number of features used to build the SSH model is extremely higher than the number of its samples, causing concerns with overfitting and weak generalisation.

In the present study, we combined the experimental assay data to construct a novel in silico tool called SSH2.0 for the prediction of hydrophobic interaction risk of mAbs. The tool developed in this study predicted hydrophobic interaction risk of mAbs by using only the amino acid sequence. Compared with the previous version, SSH2.0 was trained with new features that were optimised using a new feature selection method. Overall, SSH2.0 was superior to the previous version in terms of performance.

## Dataset and Method

### Dataset

**FIGURE 1:**
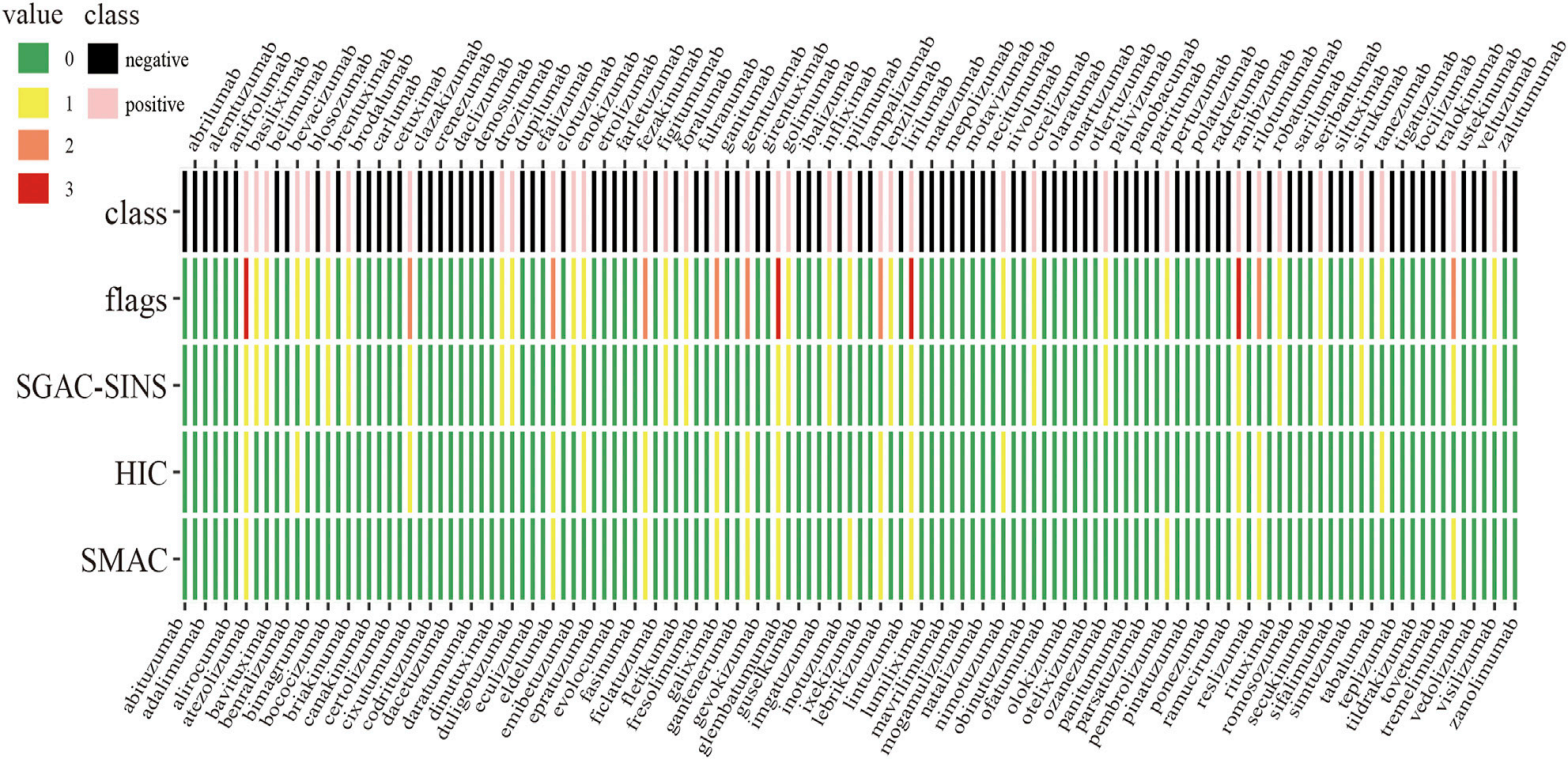
The number of hydrophobic interaction flags and the classification of antibodies.

The antibody dataset used in a study by Jain et al. (2017b) was selected in the present study. We linked the variable region in the form of “heavy chain−light chain” as the antibody sequences. The dataset comprised 137 antibody sequences (48 from approved antibodies and 89 from clinical II/III trials) and data of 12 biophysical and binding assays. Six antibody sequences with conflicting records were eliminated, resulting in inclusion of 131 antibody sequences. The assays, namely stand-up monolayer adsorption chromatography (SMAC), salt-gradient affinity-capture self-interaction nanoparticle spectroscopy (SGAC-SINS), and hydrophobic interaction chromatography (HIC), were used to determine the risk of hydrophobic interaction. A threshold of 10% was employed according to a study by Jain et al. (2017b) (Table 1). The antibody was labelled with a fault flag if one of the aforementioned three assay values exceeded the set threshold. We obtained 94 negative samples (0 flag) and 37 positive samples (25 with one flag, 8 with two flags, and four antibodies with exactly three flags). Figure 1 shows the detailed labelling of each antibody. To solve the problem of the dataset imbalance, 94 negative samples were randomly divided into three groups, with each group containing 31, 31, and 32 antibodies. Each sub-dataset (Group 1, Group 2, Group 3) was combined with positive samples to train three sub-models (SSH_a,SSH_b,SSH_c). Then, the results of the three sub-models was integrated, and an ensemble predictor was constructed using a voting strategy.

**TABLE 1:** Three experimental thresholds for evaluating the hydrophobic interaction of antibodies (Jain et al., 2017b).

Assay	Worst 10% threshold	Units (flag)
Standup monolayer adsorption chromatography (SMAC)	12.8	Retention time (min) (>)
Salt-gradient affinity-capture self-interaction nanoparticle spectroscopy (SGAC-SINS)	370	Salt concentration (mM) (<)
Hydrophobic interaction chromatography (HIC)	11.7	Retention time (min) (>)

### Feature Extraction and Selection

To construct an efficient prediction tool, appropriate feature extraction methods for transforming sequence data into numerical expressions (ideally, without distortion), in addition to a reliable benchmark data set, are crucial. Features based on sequence information such as the amino acid composition and pseudo amino acid components (He et al., 2019; Dzisoo et al., 2020; Wang et al., 2020), displayed good performance in protein and peptide classification (He et al., 2016; Li et al., 2017; Kang et al., 2019). Based on a large number of experimental results, the CKSAAGP (composition of k-spaced amino acid group pairs) (Chen et al., 2009; Chen et al., 2018) demonstrated the best performance in the present study. In the CKSAAGP encoding scheme, 20 amino acids were divided into the following five groups according to their physicochemical properties: g1: aliphatic group (GAVLMI); g2: aromatic group (FYW); g3: positive charge group (KRH); g4: negative charged group (DE); g5: uncharged group (STCPNQ) (Chen et al., 2018). Then, the frequency of amino acid group pairs separated by k residues was calculated (the default maximum value of k was set as 5). CKSAAGP can be defined as follows:

$$\left(\frac{N_{g1g1gap0}}{N_{total}},\frac{N_{g1g2gap0}}{N_{total}},\frac{N_{g1g3gap0}}{N_{total}},\ldots ,\frac{N_{g5g4gap5}}{N_{total}},\frac{N_{g5g5gap5}}{N_{total}}\right)$$

where $N_{g1g1gap0}$ represents the number of times that the composition of the residue pair g1g1 is separated by 0 amino acids in the whole protein sequence; $N_{total}$ represents the total number of k-spaced amino acid pairs. For a protein of length P, k = 0, 1, 2, 3, 4, and 5, and the values of $N_{total}$ are P-1, P-2, P-3, P-4, P-5, and P-6, respectively. CKSAAGP can be used to encode unequal length sequences.

To compare the influence of different feature extraction algorithms, we used 19 feature extraction methods on the same dataset and constructed 19 models. The feature extraction methods tested in this study are AAC, DPC, TPC, CKSAAP, DDE, GAAC, GDPC, GTPC, Moran, Geary, NMBroto, CTDC, CTDT, CTDD, CTriad, KSCTriad, SOCNumber, QSOrder, and PAAC. All feature extraction processes were performed using the iFeature (Chen et al., 2018) python package, which can be obtained from github (https://github.com/Superzchen/iFeature/).

High-dimensional small sample data usually cause the problem such as overfitting, longer training time and redundant features. In this study, an integrated method MRMD2.0 developed by He et al. (2020) was used for feature sorting and dimension reduction. MRMD2.0 represents different feature ranking with directed graph. Then the PageRank algorithm was used to obtain the new ranking. Finally, sequential forward selection (SFS) was used to select the optimal feature subset.

### Support Vector Machine Model Establishment

Owing to a high prediction accuracy and simple parameter optimisation, support vector machine (SVM) has been applied extensively in many fields such as protein−protein interactions (Romero-Molina et al., 2019), drug discovery (Patel et al., 2020), and medical image processing (Yang et al., 2019). The basic idea of SVM is to determine the hyperplane with the largest interval in the space, which can divide positive and negative samples effectively and accurately. We employed LIBSVM (Chang and Lin., 2011) to construct the SVM sub-models. Among the given four kernel functions, we chose the radial basis function (RBF) kernel to obtain the optimal kernel parameter γ and penalty parameter *C*. Three sub-models were integrated through the voting strategy. The results of the three sub-models were integrated, and an antibody was predicted to have high risk of hydrophobic interaction if it was predicted as a positive sample by at least two models.

### Performance Evaluation

Leave-one-out cross-validation (LOOCV) was adopted to assess the performance of each sub-model. One sample in the sub-dataset was used as the test set, whereas the remaining samples constituted the training set. This process was repeated N times (where N is the number of samples). Eventually, the average prediction accuracy was considered as the final accuracy of the sub-model. The performance of the prediction models was evaluated using the common indicators, namely sensitivity (Sn), specificity (Sp), accuracy (ACC), and Matthews correlation coefficient (MCC). MCC is a relatively balanced indicator for prediction that is mainly used to measure dichotomy. It comprehensively considers TP, TN, FP, and FN, which can avoid sample imbalance deviation. These indicators can be expressed as follows:

$$Sn=\;\frac{TP}{TP+FN}$$

$$Sp=\;\frac{TN}{TN+FP}$$

$$ACC=\;\frac{TN+TP}{TP+FN+TN+FP}$$

$$MCC=\;\frac{TN\times TP-FP\times FN}{\sqrt{\left(TP+FP\right)\left(TP+FN\right)\left(TN+FP\right)\left(TN+FN\right)}}$$

where TP and TN represent the number of positive data and negative data, respectively, that were predicted correctly, whereas FP and FN represent the number of positive data and negative data, respectively, that were erroneously predicted. In addition, AUC (area under the ROC curve) was used to illustrate the performance of the model. ROC curve is a TPR vs FPR plot that illustrates the diagnostic ability of a binary classifier system as its discrimination threshold is varied. AUC value ranges from 0 to 1. A model whose prediction efficiency is 100% has an AUC value of 1.

### Developability Index (DI) Calculation

The developability index (DI) of each antibody in a study by Jain et al. (2017b) was computed using BIOVIA Discovery Studio 2019 (BIOINFORMATICS SOCIETY OF SICHUAN PROVINCE) with the default parameters pH = 6 and *β* = 0.05. The crystal structure of each antibody, if available, was downloaded from the PDB database. For the antibodies whose crystal structure was not available, we performed homology modelling to build their structure. Spearman rank correlation was used to explore the correlation between DI and 12 experiment assays (Jain et al., 2017b). Statistical analysis was performed with R4.1.0.

### Online Web Service

To facilitate the use of researchers, a user-friendly web server was developed. We used HTML, CSS, PHP, JavaScript to write the interface script for web service. The data processing process script was written using *Python*.

## Results

### Feature Selection Based on CKSAAGP

**FIGURE 2:**
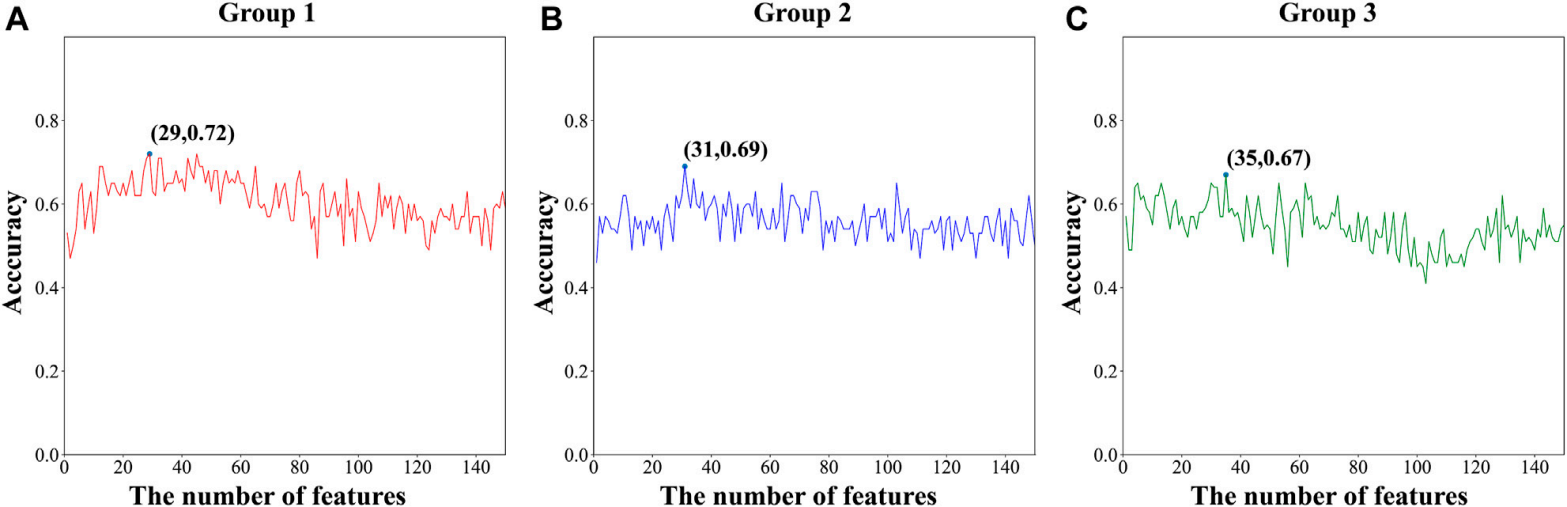
The ACC of different feature numbers during the sequential forward selection process of three sub-datasets (Group 1, Group 2, Group 3).

From a total of 150 features, the optimal feature was selected using MRMD2.0. Finally, the three sub-datasets were respectively composed of 29, 31, and 35 features. Figure 2 shows the variation of ACC with feature number during the sequential forward selection process. After feature selection, AUC was increased by at least 12% (Group 3) compared with the previous value. The prediction accuracy of the model increased with a decrease in the number of features. The small number of features also reduced the computational cost, model complexity, and the risk of overfitting. The feature dimensions of the sub-datasets were all reduced by more than 70%, which demonstrated that the performance of MRMD2.0 was excellent.

### Model Evaluation

**FIGURE 3:**
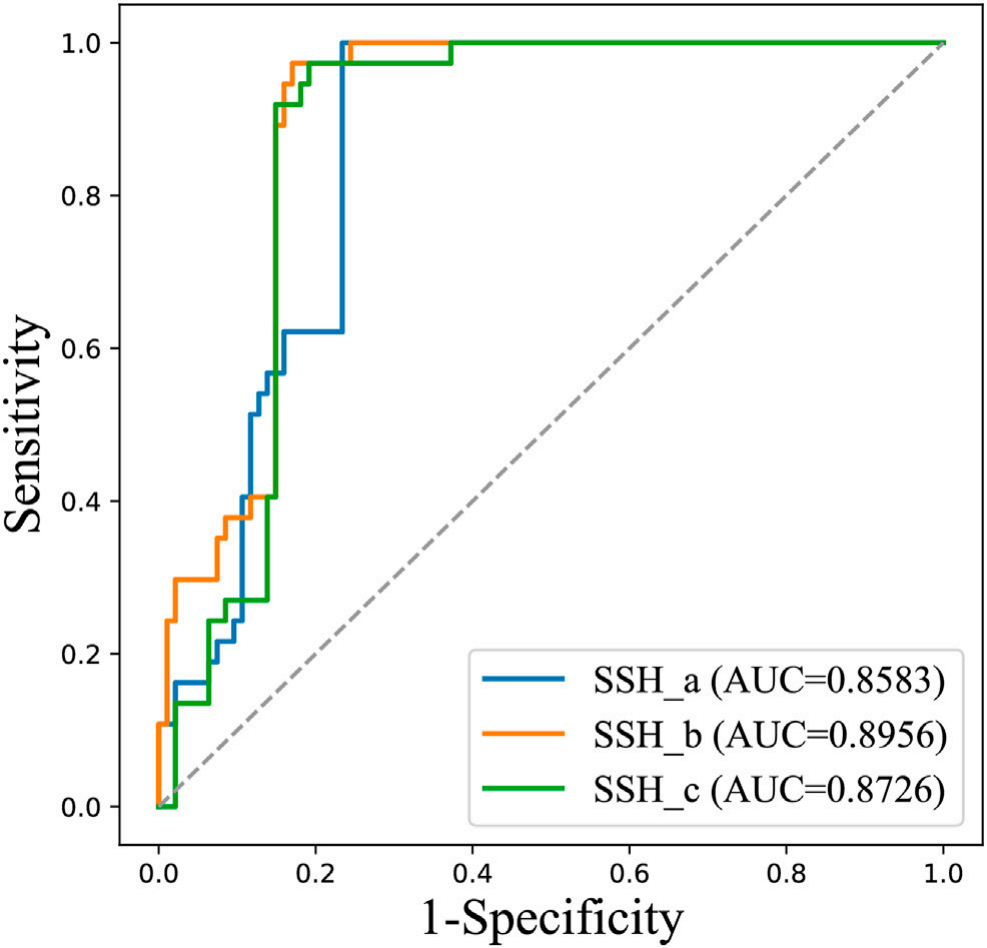
The ROC curves of three sub-models for predicting all 131 antibodies.

We trained three SVM sub-models based on LOOCV using the optimal features. As shown in Table 2, the accuracy rates of SSH_a, SSH_b and SSH_c for the prediction of antibody hydrophobic interaction were 80.88, 77.94 and 75.36% respectively. By considering all samples as input of each sub-model, we obtained three prediction results. To visually demonstrate the ability of each sub-model to predict the hydrophobic interaction, a receiver operating characteristics (ROC) curve was drawn (Figure 3). The AUC value of SSH_a, SSH_b and SSH_c reached 0.8583, 0.8956, and 0.8726, respectively. According to the aforementioned analysis, an ensemble model called SSH2.0 was constructed based on voting strategy. The sensitivity of the ensemble model was 100.00%, indicating that SSH2.0 can correctly identify all antibodies with a risk of hydrophobic interaction (Table 2).

**TABLE 2:** The prediction performance of three sub-models evaluated through leave-one-out cross-validation and that of the ensemble model evaluated through voting strategy.

Model	Sn(%)	Sp (%)	ACC(%)	MCC	AUC
SSH_a	81.08	80.64	80.88	0.6159	0.8086
SSH_b	81.08	74.19	77.94	0.5544	0.7763
SSH_c	78.37	71.87	75.36	0.5038	0.7513
SSH2.0	100.00	77.66	83.97	0.7039	0.8883

### Comparison of Different Feature Extraction Methods

**FIGURE 4:**
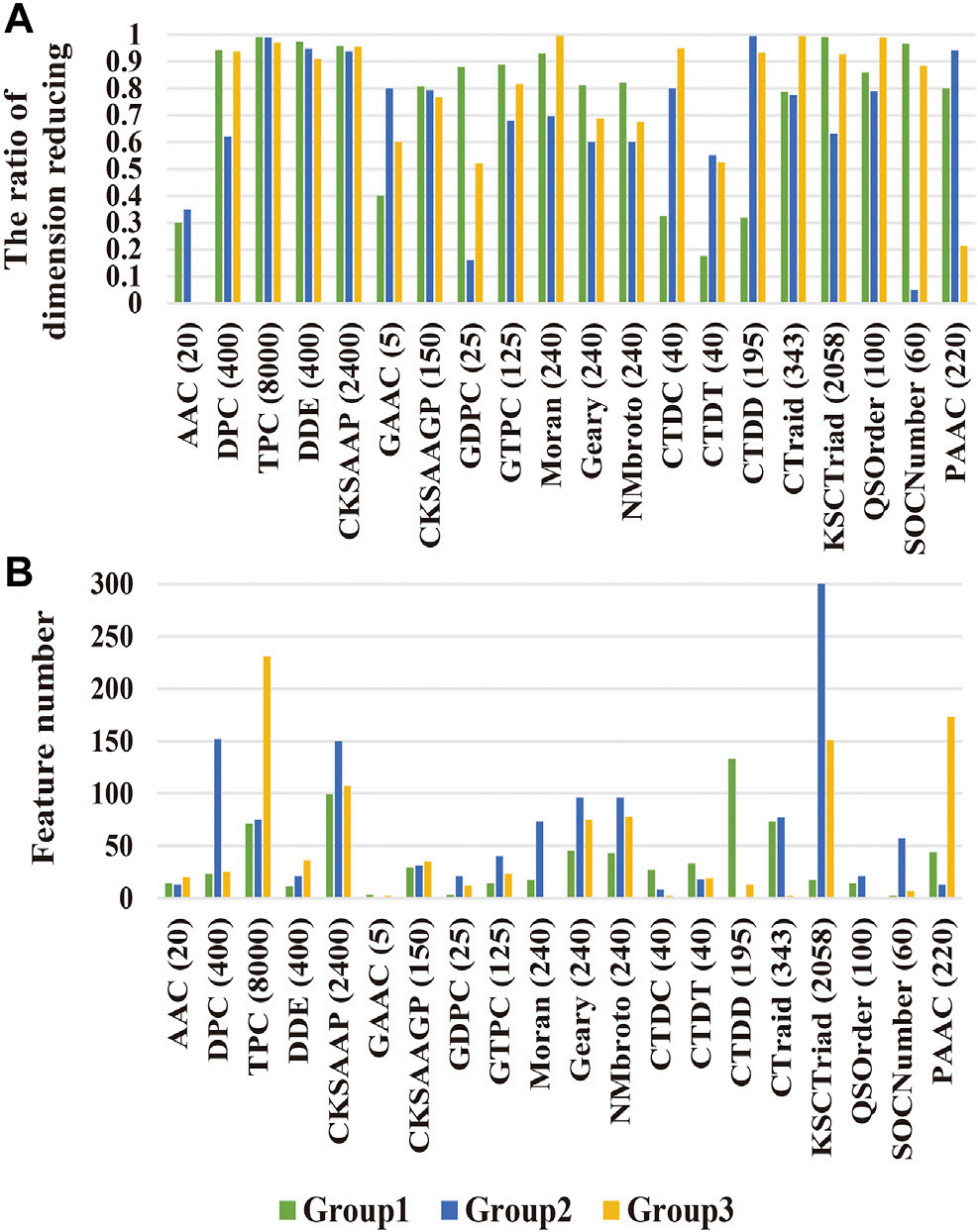
Analysis of MEMD2.0 dimensionality reduction results. **(A)** The reduced ratio and **(B)** the number of features in the dimension of three sub-datasets. The numbers in parentheses are the original feature numbers of various feature extraction algorithm.

To comprehensively evaluate the effect of the CKSAAGP algorithm, we compared it with the other 19 feature extraction algorithms. Figure 4 shows the feature dimension and dimension decline percentage obtained using all 20 algorithms after the reduction of MRMD2.0. The dimensions of multiple methods were reduced by more than 70%; however, the number of features varied among the three sub-datasets. For example, the number of TPC features decreased from 8,000 to 71 and 75 in Group 1 and Group 2, respectively, whereas that in Group 3 was 231. These results indicated that all feature extraction algorithms were affected by the samples, whereas CKSAAGP had smaller feature dimensions in all three sub-datasets with smaller variance, which was relatively robust. Furthermore, we assessed the ensemble model based on all 20 algorithms. As shown in Table 3, although the sensitivity of multiple features had reached 100%, CKSAAGP showed the highest specificity, accuracy, MCC and AUC of 77.66%, 83.97%, 0.7093, and 0.8883, respectively. Taken together, CKSAAGP was the most proper feature type for this problem, considering feature dimensions and the performance of sub-models and ensemble model.

**TABLE 3:** The prediction performance of the ensemble model based on 20 feature extraction algorithms.

Feature	Sn (%)	Sp(%)	ACC(%)	MCC	AUC
CKSAAGP	100.00	77.66	83.97	0.7039	0.8883
CTriad	100.00	75.53	82.44	0.6825	0.8777
DPC	100.00	72.34	80.15	0.6518	0.8617
TPC	100.00	71.28	79.39	0.6419	0.8564
AAC	100.00	70.21	78.63	0.6322	0.8511
CKSAAP	100.00	69.15	77.86	0.6226	0.8457
NMBroto	97.30	69.15	77.10	0.5983	0.8322
DDE	100.00	65.96	75.57	0.5947	0.8298
GTPC	100.00	63.83	74.05	0.5767	0.8191
CTDC	97.30	65.96	74.81	0.5699	0.8163
CTDT	91.89	63.83	71.76	0.5021	0.7786
CTDD	97.30	56.38	67.94	0.4910	0.7684
Geary	100.00	53.19	66.41	0.4929	0.7660
SOCNumber	100.00	52.13	65.65	0.4850	0.7606
Moran	100.00	50.00	64.12	0.4693	0.7500
QSOrder	83.78	60.64	67.18	0.4003	0.7221
KSCTriad	100.00	40.43	57.25	0.4010	0.7021
GAAC	75.68	62.77	66.41	0.3464	0.6922
GDPC	100.00	30.85	50.38	0.3345	0.6543
PAAC	100.00	0.00	28.24	0.0000	0.5000

### CKSAAGP Features That Closely Related to the Hydrophobic Interaction

The properties of amino acid side chains are closely related to the structure and function of proteins. The nonpolar amino acids (aliphatic, and aromatic amino acids) are usually hydrophobic. Conversely, the polar amino acids (positively and negatively charged and uncharged amino acids) are hydrophilic. Among all the features in models, aliphatic. aliphatic.gap5, aromatic. aliphatic.gap3, negativecharger. aliphatic.gap1 were present in all sub-models, and only one of these features, namely aromatic. aliphatic.gap3, was in the top 10 features (Table 4). The binding of nonpolar amino acids with strong hydrophobicity increases the hydrophobicity of the protein. Interestingly, as shown in Table 4, the combination “polar + nonpolar” appeared frequently, which indicated that a polar amino acid and a nonpolar amino acid are separated by several amino acids in space that probably enhances the hydrophobicity of the protein, although a single polar amino acid is hydrophilic. In summary, if the CKSAAGP features listed in Table 4 appear frequently in an antibody sequence, the antibody should be excluded from early development.

**TABLE 4:** The top 10 CKSAAGP features of three sub-models. The features marked in red indicate that they exist in at least two sub-models (neg: negative charged group; pos: positive charge group).

SSH_a	SSH_b	SSH_c
aromatic.uncharge.gap0	aromatic.aliphatic.gap1	aliphatic.pos.gap0
uncharge.uncharge.gap0	aliphatic.neg.gap3	uncharge.aliphatic.gap4
aromatic.aliphatic.gap3	pos.aliphatic.gap2	uncharge.aromatic.gap2
pos.neg.gap0	uncharge.uncharge.gap2	neg.aromatic.gap5
aliphatic.aromatic.gap5	aliphatic.pos.gap0	pos.uncharge.gap5
uncharge.uncharge.gap2	neg.uncharge.gap4	aliphatic.uncharge.gap5
pos.uncharge.gap0	aliphatic.aromatic.gap5	aromatic.aliphatic.gap3
pos.uncharge.gap4	neg.aliphatic.gap2	aliphatic.uncharge.gap1
neg.pos.gap2	aromatic.uncharge.gap2	aliphatic.aliphatic.gap2
aliphatic.uncharge.gap5	aromatic.pos.gap1	neg.neg.gap3

### Comparison Between the Previously Constructed SSH Model and DI Computational Tool

In our previous study, Dzisoo et al. (2020) provided a web-server named SSH based on TPC features to predict the hydrophobic interaction risk of mAbs. However, the number of features in SSH was far more than the number of samples, which indicated the probability of overfitting. In this study, we optimized the feature extraction algorithm and feature selection method to maintain the prediction accuracy with fewer features. We uniformly defined sensitivity as the ability to identify samples with hydrophobic interaction risk. As shown in Table 5, the number of each SSH sub-model features was more than 300, whereas the number of samples used for training was < 70. After using the CKSAAGP feature scheme and MRMD2.0 feature selection algorithm, the number of features in SSH2.0 reduced to one-tenth that of SSH. Although the ACC and AUC of the ensemble model decreased by 7.26% and 0.0737, respectively, we paid more attention to the performance to identify defective samples. The sensitivity of SSH2.0 reached 100.00%, which was 16.70% higher than that of SSH.

**TABLE 5:** Comparison of the feature and performance between SSH2.0 and SSH.

Model	Feature	Feature extraction method	Feature number of sub-models	Sn(%)	Sp(%)	ACC(%)	AUC
SSH	TPC	f -scores	313,315,315	84.30	96.39	91.23	0.9620
SSH2.0	CKSAAGP	MRMD2.0	29,31,35	100.00	77.66	83.97	0.8883

**FIGURE 5:**
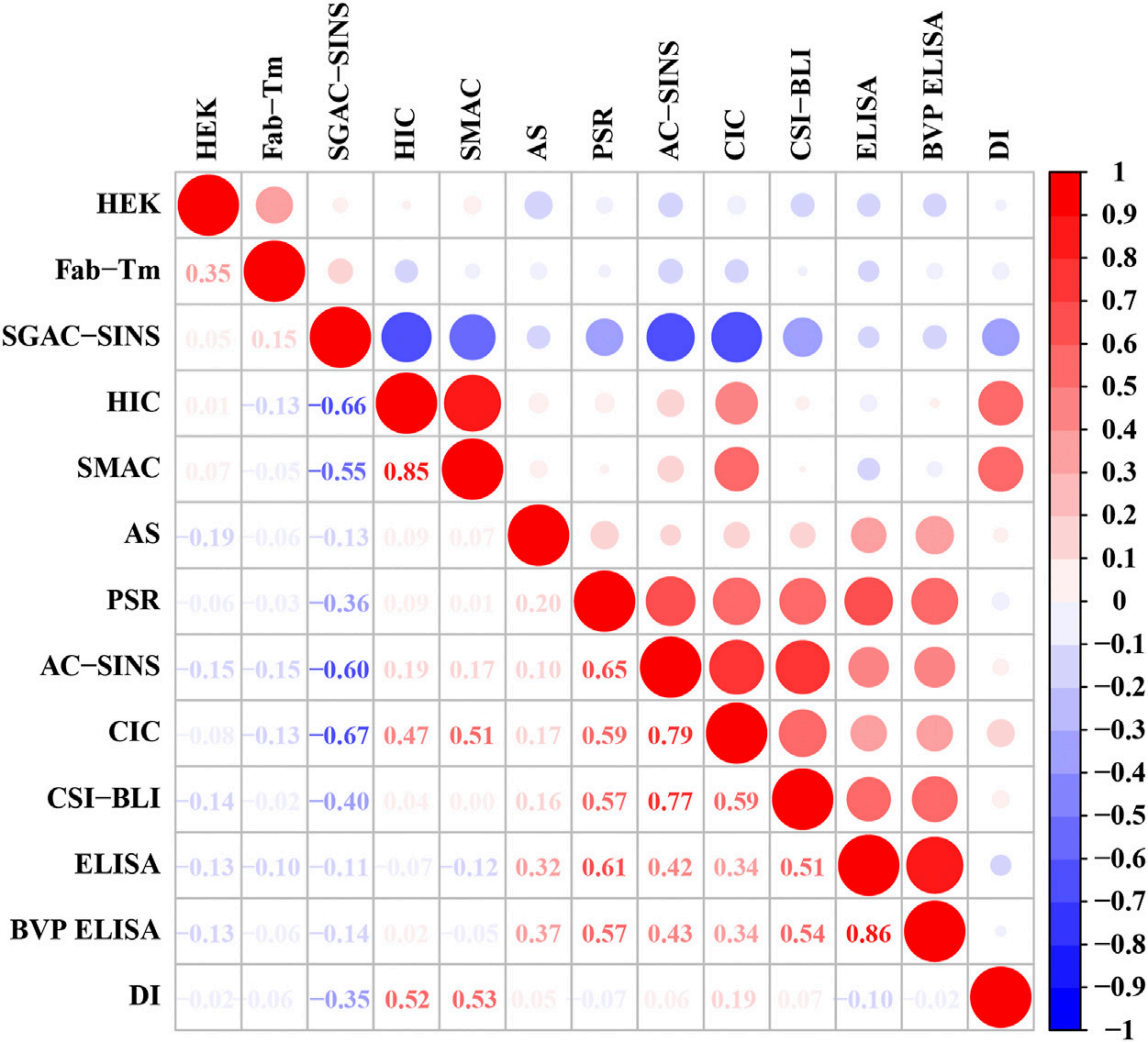
Correlation coefficient matrix of DI and 12 experimental assays. The lower triangle shows the spearman correlation coefficients, and the upper triangle represents the corresponding correlation values. The radius of the circles is proportional to the magnitude of the correlation coefficient. Red represents a positive correlation, and blue represents a negative correlation.

DI is another widely employed tool for assessing the aggregation propensity of proteins (Lauer et al., 2012). We performed the Spearman rank correlation test to explore the correlation between DI and 12 experimental assays. Surprisingly, the three most relevant assays were SMAC, SGAC-SINS and HIC (Figure 5), which we used to assess the hydrophobic interaction risk of mAbs in the current study. The result confirmed that protein aggregation is mainly driven by hydrophobic interactions (Hebditch et al., 2019). According to the methods based on the experimental data presented by Jain et al. (2017b), 37 antibodies were flagged with hydrophobic interaction warnings. We used this as the gold standard. Because high DI values correspond to low developability (Lauer et al., 2012), we sorted all the antibodies according to the descending order of their DI values. The top 37 antibodies with high DI values were predicted to have the hydrophobic interaction risk. However, the prediction performance of the DI method was inferior to that of SSH2.0. The accuracy rates of SSH2.0 and DI were 83.97 and 61.83%, respectively. The results suggest that owing to the low prediction accuracy, the application of DI to a screening platform would lead to many antibodies with a high aggregation risk being incorrectly selected.

### Web-Server Guidance

**FIGURE 6:**
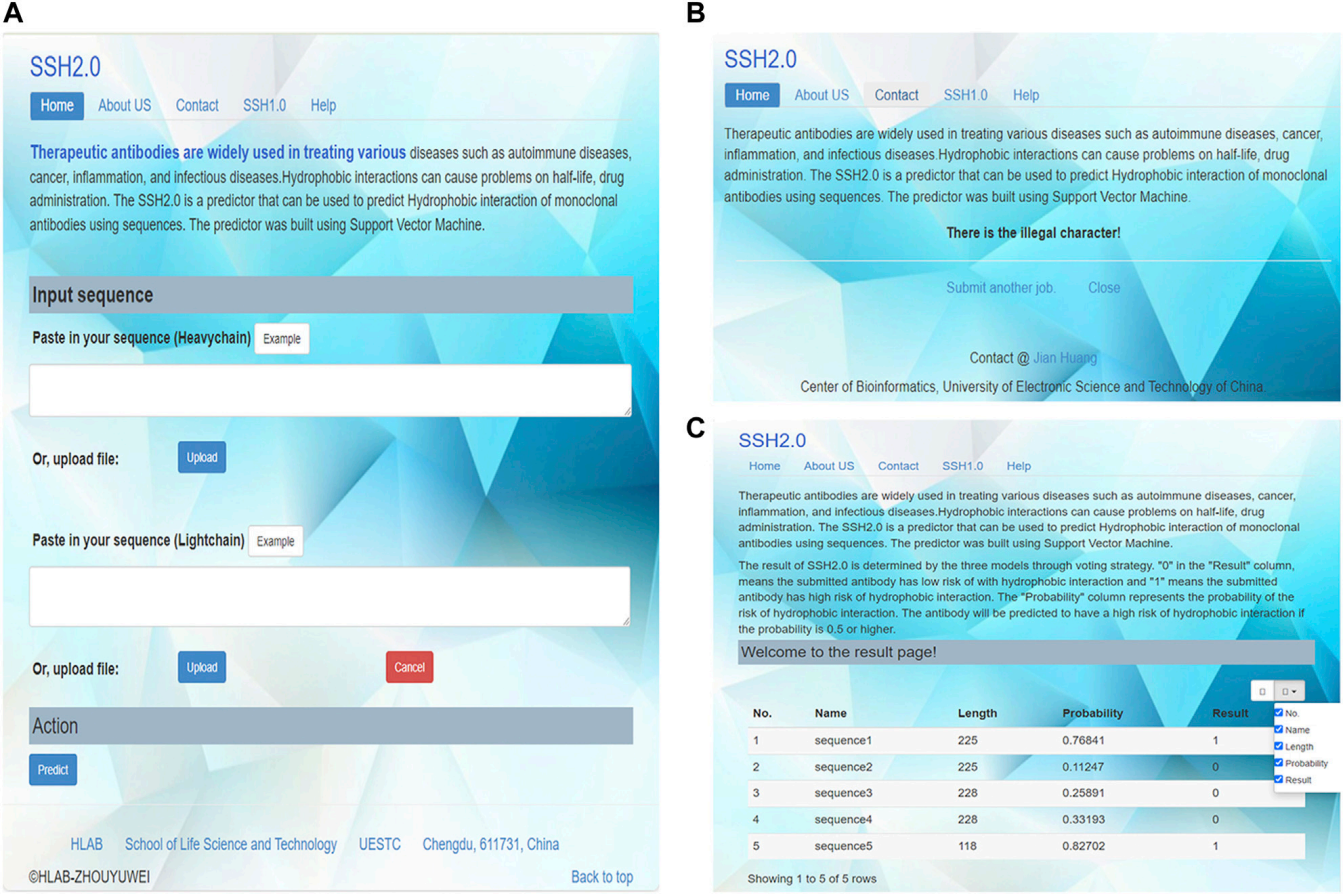
Screenshots of the SSH2.0 web server. **(A)**Homepage of the SSH2.0 web server. **(B)** If illegal characters appear in the input sequence, click “predict” bottom and a prompt page will pop up, The prompt page showing “There is the illegal character!”. Users can click “submit another job.” to return to the home page and resubmit the sequence. **(C)** Result display page. “1” in the “Result” column denotes that the submitted antibody candidate exhibits a high risk of hydrophobic interaction and should be excluded from the development pipeline. The “Probability” column represents the probability of the risk of hydrophobic interaction. The antibody will be predicted to have a high risk of hydrophobic interaction if the probability is 0.5 or higher. The result table can be sorted according to each column, and a custom display box allows users to select and display specific information as needed.

To serve the relevant researchers, we established a user-friendly web server for the prediction of hydrophobic interaction risk of mAbs. The server is freely accessible at http://i.uestc.edu.cn/SSH2/. The homepage of SSH2.0 is shown in Figure 6A. The variable region sequences of heavy chains and light chains were input separately. Because some antibodies only have one chain, the input consisting of single heavy or light chain were allowed. The submitted antibody sequences were in the FASTA format. The AbRSA tool can help in antibody numbering and CDR (complementarity-determining region) delimiting (Li et al., 2019). SSH2.0 allowed the detection of illegal characters, and only 20 common amino acids were found to be legal for sequence input. Illegal characters such as B, J, O, U, X, Z and the numbers 1–9 were forbidden (Figure 6B). Figure 6C shows the prediction results.

## Discussion

The developability assessment is performed mainly to evaluate the biochemical and biophysical properties of mAbs and to select the lead antibody with ideal efficacy, safety, pharmacokinetic characteristics, and physicochemical characteristics to meet the technical requirements of the production and preparation processes (Xu et al., 2019). Various experimental strategies have been used to identify the unfavourable physicochemical properties of mAbs. However, experimental assays are time-consuming, expensive, and laborious. Computational methods can provide rapid and highly economic evaluation results and thus are expected to promote the development of antibodies (Krawczyk et al., 2017). DI is a well-known in silico tool for assessing the aggregation propensity of therapeutic antibodies and it is based on the principles that protein aggregation is mainly driven by hydrophobic interactions. Regretfully, this tool relies on the antibody structure and runs slowly. Moreover, it is an expensive tool, which makes its application limited for high-throughput screening of mAbs at the early developmental stage.

Currently, data mining and machine learning are widely applied in antibody development research (Dzisoo et al., 2021). Lecerf et al. (2019) confirmed that the sequence characteristics of the antibody variable region can determine the physicochemical properties of therapeutic antibodies. Obrezanova et al. (2015) constructed a model to predict the aggregation propensity based on the antibody sequence, and the AUC of the best AdaBoost model reached 0.76. Furthermore, Jain et al. (2017a) constructed a model to predict the solvent-accessible surface area of each amino acid residue in the variable region based on the amino acid sequence of the antibody and predicted the hydrophobic interaction of antibodies through simple logistic regression. However, aforementioned tools do not provide available model or sever.

The hydrophobic interaction prediction model constructed in the present study was trained on sequence only and eliminated the requirement of 3D protein structure, thereby saving the computation resources. The high sensitivity usually corresponds to the low specificity. The sensitivity of SSH2.0 reached 100.00%, which indicated that the SSH2.0 prediction result may have more false positives. However, the high sensitivity of SSH2.0 is acceptable or even preferred because the main purpose of this tool is to exclude antibodies with a risk of unfavourable hydrophobic interactions. In addition, after the step of modern mAb discovery, usually tens of thousands of therapeutic antibody candidates remain to be evaluated, and the presence of even more false positives in SSH2.0 prediction results is affordable. In summary, we propose that SSH2.0 is an efficient model for predicting the hydrophobic interaction risk of mAbs.

The hydrophobic interaction risk predictor SSH2.0 constructed in this study for therapeutic mAb development is a powerful tool for selection of the antibody drug candidates with a high risk of hydrophobic interaction. This free tool based on the antibody sequence might be a better and faster alternative to the existing DI computational tool. We expect that the newer version of this tool can be used to identify reasonable mutants with a decreased risk of hydrophobic interaction. Because the number of proven therapeutic antibodies is limited, and the experiment assays vary across batches, we also expect the tool can be assessed by an independent dataset in future.

## Conclusion

In this study, we developed SSH2.0, a SVM-based ensemble model trained with CKSAAGP features, for predicting the hydrophobic interaction risk of therapeutic mAbs. Compared with our previous model SSH and the widely used DI tool, SSH2.0 may be a better and robust predictor that achieved the maximum sensitivity of 100.00%, and ACC and AUC of 83.97 and 88.83%, respectively. We also developed a user-friendly web server, which is freely available at http://i.uestc.edu.cn/SSH2/. This tool offers a high-throughput and efficient assessment of the developability of antibodies from the perspective of hydrophobic interaction risk.

## Data Availability

Publicly available datasets were analyzed in this study. This data can be found here: https://www.pnas.org/content/114/5/944/tab-figures-data.
